# Habit-learning and decision-making circuits are susceptible to glycemic variability in type 2 diabetes: a longitudinal study

**DOI:** 10.3389/fnins.2025.1430185

**Published:** 2025-07-09

**Authors:** Carolina Moreno, Otília C. d’Almeida, Joana Crisóstomo, Nádia Canário, Leonor Gomes, Miguel Castelo-Branco

**Affiliations:** ^1^Faculty of Medicine, University of Coimbra, Coimbra, Portugal; ^2^Department of Endocrinology, Diabetes and Metabolism, Hospitais da Universidade de Coimbra, ULS Coimbra, Coimbra, Portugal; ^3^CIBIT, Coimbra Institute for Biomedical Imaging and Translational Research, Institute of Nuclear Sciences Applied to Health (ICNAS), University of Coimbra, Coimbra, Portugal

**Keywords:** type 2 diabetes, brain volume, MRI, glycemic variability, cerebral atrophy, longitudinal

## Abstract

**Objective:**

Type 2 diabetes mellitus (T2DM) is associated with lower gray matter (GM) volumes. However, little is known about the impact of glycemic control on brain atrophy, especially in highly susceptible regions. Therefore, we aim to identify the effect of glycemic variability (GV) on long-term changes in brain volume among individuals with T2DM.

**Methods:**

A longitudinal clinical, biochemical, and imaging assessment was conducted at a baseline visit on 170 individuals (85 with T2DM), from which 29 (15 with T2DM) were evaluated at a 7-year follow-up visit. Brain regional volumes were evaluated with 3 T MRI, using the FreeSurfer 7 longitudinal pipeline. GV metrics such as SD, M-value, MAG (mean absolute glucose change), MAGE (mean amplitude of glycemic excursion), and CoV (coefficient of variation) were calculated in both visits.

**Results:**

Statistically significant negative correlations between GV metrics and symmetrized percent change (SPC) of GM volumes were found in specific cortical and subcortical regions of individuals with T2DM. MAGE was correlated with regionally specific atrophy on the temporal lobe (*r* = −0.63, *p* = 0.021), insula (*ρ* = −0.62, *p* = 0.022), thalamus (*r* = −0.64; *p* = 0.024), hippocampus (*r* = −0.59; *p* = 0.034), and putamen (*ρ* = −0.65, *p* = 0.017). Concerning the hippocampal subregions, the presubiculum was significantly correlated with MAGE (*r* = −0.73; *p* = 0.005). Baseline GV was consistently associated with temporal lobe SPC. Linear regression analysis showed that, for each increase of 1 mmol/L in MAGE value, the SPC of the temporal lobe decreases on average by 1.2% (higher atrophy rate).

**Conclusion:**

The relationship between longitudinal GM atrophy and GV has a regionally specific pattern, suggesting localized brain susceptibility to intra-daily glucose fluctuations. Negative correlations between GV metrics and SPC volume of regions involved in habit-learning, decision-making, and memory highlight GV as a mediator of the neural impact of T2DM on the reward prediction-error circuits.

## 1 Introduction

Diabetes mellitus is associated with various changes in brain function and structure (Biessels and Reijmer, 2014). Brain atrophy, characterized by lower total and regional gray matter (GM) volumes, has consistently been reported in patients with type 2 diabetes (T2DM), particularly among older adults with cognitive dysfunction (Geijselaers et al., 2015). With the global epidemic of T2DM and the growing aging population (Khan et al., 2019), a focus on diabetes brain health might have an additional impact on delaying or even preventing diabetes-related mental health conditions.

Previous longitudinal studies have shown relatively homogeneous results regarding the accelerated progression of brain atrophy in individuals with type 2 diabetes compared to controls (de Bresser et al., 2010; van Elderen et al., 2010; Brundel et al., 2014; Espeland et al., 2016; Callisaya et al., 2019). However, there is conflicting evidence on the diabetes-related risk factors that contribute to structural changes in the brain. Van Elderen et al. found a correlation between brain volume loss with fasting glucose levels and insulin therapy (van Elderen et al., 2010). Furthermore, de Bresser et al. demonstrated that increasing age and hypertension were associated with a greater progression of brain atrophy (de Bresser et al., 2010). However, Brundel et al., using ultra-high-field MRI, did not find any differences in the burden of microvascular lesions between diabetic patients and control groups, nor did they find any significant relationships with cognitive testing (Brundel et al., 2014). By analyzing data from the ‘Second Manifestations of ARTerial disease-Magnetic Resonance’ (SMART-MR) study, Kooistra et al. found an increased rate of brain atrophy and vascular lesion load in diabetes individuals with symptomatic atherosclerotic disease only slightly exceeding controls, but with no changes in cognitive performance over time (Kooistra et al., 2013).

Regarding the distribution of brain atrophy throughout the brain, longitudinal data are missing; however, it appears that there is a variable impact across brain areas, with the temporal lobe and hippocampus showing more susceptibility to atrophy (Moheet et al., 2015; Zhang et al., 2022). This regional vulnerability is noteworthy, as connectivity between the temporal lobe and subcortical regions relates to behavioral regulation and cognitive control regarding responses to emotional cues (D’Ostilio et al., 2012; Heller et al., 2016). The reward dopaminergic system, encompassing mesolimbic and mesocortical pathways, might be involved in dysmetabolic conditions, particularly through disruption of insulin signaling. Animal and human studies have reported insulin as a key regulator of dopamine turnover, as reduced insulin-sensitivity correlates with reduced endogenous dopamine levels, and the apoptotic processes of dopaminergic neurons are influenced by insulin (Caravaggio et al., 2015; Fiory et al., 2019).

In the ‘Leukoaraiosis and disability in the elderly study’ (LADIS), Korf et al. showed an association between medial temporal lobe atrophy and diabetes, independently of the amount of small vessel disease (Korf et al., 2007). Hayashi et al. reported that hippocampal and whole brain atrophy was more frequent in older individuals with T2DM than in controls (Hayashi et al., 2011). Den Heijer et al. found that T2DM subjects had greater hippocampal and amygdalar atrophy compared with controls, regardless of vascular pathology (den Heijer et al., 2003). However, in a large cross-sectional study, Wisse et al. showed that patients with T2DM had greater brain atrophy but not hippocampal atrophy compared to controls (Wisse et al., 2014). Furthermore, no associations were found between brain volume and HbA1c or memory outcomes. Consistent clinical correlates of regional brain atrophy in diabetes are lacking, and the mechanisms underlying the central nervous system microstructural abnormalities are still unclear. With the well-established microvascular cerebral dysfunction in diabetes (van Sloten et al., 2020), rapid glucose fluctuations might directly affect brain structure and function. An accumulation of aberrant metabolites, depletion of metabolic cofactors, and incremental oxidative stress has been described as a consequence of rapid glycemic peaks, leading to neuroinflammation, neuronal dysfunction, and apoptosis (Sima et al., 2004; Van Dyken and Lacoste, 2018).

Glycemic variability is a valuable tool for diabetes management in the clinical setting, since its metrics that represent short-term or long-term glycemic excursions are independent risk factors for diabetes complications (Ceriello et al., 2019). In cross-sectional studies, glycemic variability has been negatively associated with cognitive function (Zhong, 2012; Rizzo et al., 2010; Lee et al., 2022). Furthermore, time-specific multi-scale glycemic variability may contribute to cognitive impairment and GM atrophy as indirectly estimated from statistical parametric mapping (SPM) using the LONI Probabilistic Brain Atlas in a longitudinal study of older adults with and without T2DM (Cui et al., 2014). Future prospective data might finally establish glycemic variability as a key player in diabetes-related brain outcomes.

In the present study, a longitudinal case–control investigation was conducted to explore regional cerebral correlates of T2DM brain structural changes in a 7-year follow-up. We hypothesized that relationships between short-term glycemic variability metrics and GM volume could be independent from long-term glycemic control, e.g., HbA1c, clarifying the role of glycemic variability on brain structure of T2DM.

## 2 Methods

### 2.1 Study design

Individuals with T2DM were recruited from the Endocrinology Department, along with a control group from the local community, during two years (2012–2013), for the baseline evaluation (Visit 0). All participants were contacted by telephone for a follow-up assessment (Visit 1) from 2019 to 2020. This study was approved by the local Ethics Committee and followed the tenets of the Declaration of Helsinki. Written informed consent was obtained from all participants after research procedures had been fully explained during both visits.

### 2.2 Eligibility criteria

All participants fulfilled the inclusion criteria: age between 45 and 75 years, T2DM confirmed by the 2019 World Health Organization (WHO) (WHO, 2019), with determination of fasting glucose levels, HbA1c, and absence of diabetes auto-antibodies (T2DM group) or exclusion of T2DM according to the same criteria (control group). In all participants, we ascertained the absence of the following exclusion criteria: history of neurological or psychiatric disease, dementia or cognitive impairment, active malignancy, inflammatory disease, chronic drug or alcohol dependence, or severe visual impairment. The eligibility criteria were reviewed and confirmed in the follow-up assessment (Visit 1). Patients with previous cerebrovascular accident or other cortical vascular pathology were excluded, as well as participants with cognitive decline or incomplete MRI protocol or low-quality criteria in any of the visits.

### 2.3 Clinical evaluation, laboratory assessments, and glycemic variability metrics

In both visits, all participants were submitted to a thorough clinical examination performed by a team of physicians, which included personal medical history, complete physical exam with ophthalmology assessment (retinal fundus photographs and optical coherence tomography), diagnosis and characterization of other micro/macrovascular complications (diabetic peripheral neuropathy defined using the Toronto Consensus Statement (Tesfaye et al., 2010), previous history of peripheral artery occlusion or myocardial infarction) and neurophysiological testing. Blood and urine samples were collected to determine inclusion criteria, disease status, and diabetic nephropathy staging (urinary albumin-to-creatinine ratio).

Glycemic variability metrics were calculated with EasyGV® software (available free for noncommercial use at www.easygv.co.uk) using 7-point blood glucose profiles. Blood glucose was obtained throughout the day, namely, fasting (07:00), post-breakfast (08:30), pre-lunch (12:00), post-lunch (13:30), pre-supper (19:00), post-supper (20:30), and nighttime (24:00) for 72 h, as described elsewhere (Kilpatrick et al., 2006; Siegelaar et al., 2011). In Visit 0, the blood glucose measurements were performed by the nursing staff. In the follow-up assessment, patients self-reported the 7-point blood glucose measurements for 24 h on 3 consecutive days before the visit date. We calculated the short-term glycemic variability metrics feasible with 72-h data: SD (representation of dispersion from average glucose), M-value (measure of the glucose excursions in comparison with an ideal glucose default value of 6.66 mmol/L), MAG (mean absolute glucose change per unit of time), MAGE (mean amplitude of glycemic excursions), and CoV (percentage of coefficient of variation for glucose) (Hill et al., 2011).

### 2.4 Magnetic resonance imaging procedures

All participants underwent an MRI protocol on a 3T Tim Trio scanner (Siemens, Germany) with a 12-channel birdcage head coil, at baseline. A subgroup underwent a follow-up data acquisition in a 3T Magnetom Prisma fit scanner (Siemens, Germany), equipped with a 20-channel birdcage head coil. The MRI protocol included a high-resolution T1-weighted anatomical image using a 3D Magnetization Prepared Rapid Acquisition Gradient Echo (MPRAGE) sequence (baseline: TR/TE/TI = 2530/3.42/1100 ms; follow-up: TR/TE/TI, 2530/3.5/1100 ms; FA = 7°; FoV = 256 × 256 mm^2^; 176 slices with 1 mm^3^ isotropic voxel size). All acquisitions were performed at the Institute of Nuclear Sciences Applied to Health (ICNAS) of the University of Coimbra.

### 2.5 Magnetic resonance imaging analysis

Structural MRI data were processed using the FreeSurfer longitudinal pipeline (version 7.0, http://surfer.nmr.mgh.harvard.edu/) on a Linux (CentOS 6) platform following the standard “recon-all” stream (technical details of the procedures are described online, Freesurfer website). The longitudinal approach (Reuter et al., 2012) is a more robust and sensitive strategy considering that the scans are from the same individual at different timepoints. Accordingly, the initialization of several pre-processing steps is based on common information from an unbiased within-subject template, controlling, at least partially, for the within-individual variability bias (Reuter et al., 2012; Iglesias et al., 2016). The recon pipeline included bias field and motion correction, automated Talairach transformation, removal of non-brain tissue, tissue intensity normalization, subcortical structures segmentation and labeling with a non-linear warping atlas, gray/white matter boundary tessellation, and topology correction for surface reconstruction and cortical parcellation (Dale et al., 1999; Fischl et al., 1999). Subcortical regions-of-interest (ROI) segmentation was based on the Automatic Segmentation (ASEG) atlas (Fischl et al., 2002), and the cortical ROI parcellation was based on the Desikan-Killiany-Tourville (DKT40) neuroanatomical atlas (Klein and Tourville, 2012). Data were visually screened for soft failures, namely in skull stripping, white matter segmentation, intensity normalization, and pial surface misplacement, and manually corrected for major errors, when appropriate. Additional FreeSurfer hippocampal subregions were analyzed using a longitudinal hippocampal subfields segmentation pipeline [presubiculum, parasubiculum, subiculum, fissure, CA1, CA3, CA4, molecular layer, fimbria, molecular and granule cell layers of the dentate gyrus (GCMLDG), and the hippocampal amygdala transition area (HATA)] (Iglesias et al., 2015, 2016) that increases the accuracy of volume estimation across the two time points (Brown et al., 2020). FreeSurfer pipeline also assesses hippocampus volume in three parcellations: head, body, and tail (Brown et al., 2020).

Since laterality effects were not expected, volumes from equivalent region-of-interest ROIs from each hemisphere were averaged. Lobar volumes were also considered for analysis, as the combination of the underlying regional ROIs.

### 2.6 Statistical analysis

Statistical analysis was conducted in IBM SPSS Statistics for Windows (version 28.0), and graphics were produced using the ggplot2 package in R (version 2022.07.2). The normality of quantitative variables was evaluated using the Shapiro–Wilk test. Parametric/nonparametric testing was used accordingly. Data values in the tables represent mean ± standard deviation for normally distributed variables or median (interquartile range).

The relative change in volumes for each studied ROI of each individual was evaluated as the symmetrized percent change (SPC, %). SCP represents the change of volume (mm^3^) between baseline (V0) and follow-up (V1) concerning the average volume for both timepoints. It was calculated as follows: SPC=(V1−V0)/(0.5×(V1+V0))×100 .

Pearson’s or Spearman’s correlation coefficients were used, as appropriate, to examine associations between SPC of regional (sub)cortical areas and glycemic variability metrics – MAG, MAGE, M-value, and CoV at baseline. The Benjamini-Hochberg method was used to correct *p*-values for multiple correlations within seven main regions and glycemic variability metrics (FDR < 0.05). Other exploratory analyses were also conducted, correlating general SPC volumes and biochemical parameters.

GLM repeated measures ANOVA (rmANOVA) was used to compare the (within-subjects) SPC of the cortical volumes of brain lobes (frontal, parietal, temporal, occipital, cingulate, and insula) between T2DM and control groups (between-subjects). Similar analyses were performed independently for both subcortical ROIs and hippocampal subfields. The epsilon value was used to choose a correction approach when assumptions of sphericity were not met: Greenhouse–Geisser (GG, *ε* < 0.75), Huynh–Feldt (HF, *ε* > 0.75). For all analyses, the critical significance level for all hypothesis testing was set at 5% (two-tailed).

## 3 Results

Our original cohort comprised 170 participants evaluated at baseline (85 with T2DM and 85 healthy controls), from which 29 completed longitudinal assessment (15 with T2DM and 14 controls) after 7.1 ± 0.7 years. Two individuals from the control group were excluded at the follow-up visit for, respectively, not being compliant with the exclusion criteria and incomplete imaging protocol.

A symmetrized percentage change (SPC) was calculated to assess the longitudinal variation of regional cortical, subcortical, and hippocampal GM volumes.

Selected clinical, laboratory, and image characteristics of the study subjects at baseline (V0) and follow-up (V1) are presented in Table 1. Demographics and clinical and volumetric characteristics of the individuals with T2DM (*n* = 15) in both visits are presented in Table 2.

**Table 1:** Demographic and clinical characteristics of 27 participants (15 with type 2 diabetes; 12 controls) at baseline (V0) and at follow-up visits (V1) after 7.1 ± 0.7 years.

	Baseline (V0)		Follow-up (V1)	
	Diabetes	Control	Diabetes	Control
Age (years)	56.4 ± 8.2	53.3 ± 6.2	64.6 ± 7.7	61.4 ± 5.9
Gender (M:F)	10:5	7:5	-	-
Exercice (yes:no)	7:8	7:5	-	-
Smoking (yes:no:ex)	1:11:3	0:12:0	-	-
Drinking (yes:no)	7:8	4:8	-	-
BMI (Kg/m^2^)	27.7 ± 4.4	25.8 ± 2.4	29.5 ± 3.7**	25.7 ± 2.3**
Hypertension (yes:no)	11:4	4:8	12:3	4:8
Dyslipidemia (yes:no)	11:4	8:4	11:4	9:3
Fasting glucose (mmol/L)	10.0 ± 4.6**	5.0 ± 0.5** (*n* = 9)	8.0 ± 4.0*	5.1 ± 0.8*
HbA1c % mmol/mol	9.3 (4)*** 78.1 (43.7)***	5.6 (0.5)*** 37.7 (6.0)*** (*n* = 10)	7.9 (1.8)*** 62.8 (19.7)***	5.7 (0.5)*** 39.4 (5.5)***
Cortical volume (cm^3^)	438.2 ± 38.5	466.2 ± 52.9	433.5 ± 34.8	461.5 ± 49.8
Subcortical volume (cm^3^)	54.4 ± 4.5*	60.0 ± 7.0*	52.7 ± 4.6*	58.3 ± 7.3*

*p < 0.05; **p < 0.01; ***p < 0.001 for comparisons between groups (diabetes vs. control) at baseline (V0) and follow-up (V1). Data are presented as *n* (%), mean±SD, or median (IQR), as appropriate. For group comparisons, Student’s *t*-test (continuous variables with normal distribution), Mann–Whitney *U*-tests (continuous variables without normal distribution), and Chi-square tests (qualitative variables) were performed as appropriate. BMI, body mass index.

**Table 2:** Demographics, clinical, and volumetric characteristics of the individuals with type 2 diabetes (*n* = 15) at baseline (V0) and follow-up (V1).

	Baseline (V0)	Follow-up (V1)	*p*-value
Fasting glucose (mmol/L)	10.0 ± 4.6	8.0 ± 4.0	n.s.
HbA1c (mmol/mol) (%)	81.4 ± 33.3 9.6 ± 3.0	62.7 ± 16.7 7.9 ± 1.5	0.011
Total cholesterol (mmol/L)	4.8 (2.0)	4.0 (1.4)	n.s
LDL cholesterol (mmol/L)	3.2 (1.8)	2.1 (1.0)	0.012
eGFR (mil/min/1.73m^2^)	93.4 ± 30.5	74.8 ± 26.6	0.014
Peptide C index	0.6 (2.0)	0.1 (0.3)	<0.001
Triglyceride glucose index	9.4 ± 0.7	9.0 ± 0.8	0.034
SD	2.3 ± 1.0 ( *n* = 13)	2.4 ± 0.7	n.s.
CoV (%)	26.3 ± 7.4 *n* = 13)	27.4 ± 6.4	n.s.
MAG	0.8 ± 0.3 *n* = 13)	0.8 ± 0.3	n.s.
MAGE	2.4 ± 1.1 *n* = 13)	2.7 ± 0.9	n.s.
M-value	5.0 (7.3) *n* = 13)	5.1 (8.3)	n.s.
Duration of disease (years)	12.1 ± 7.8	19.2 ± 7.9	-
Micro/macrovascular complications (yes)	80% (*n* = 12)	80% (*n* = 12)	n.s.
Number of complications	1.6 ± 1.1	1.7 ± 1.1	n.s.
Insulin therapy (yes)	66.7% (*n* = 10)	66.7% (*n* = 10)	n.s.
Total insulin daily dose (IU)	40.8 ± 20.9	43.3 ± 21.1	n.s.
Oral antidiabetic medication			
- Metformin - DPPIV inhibitors - Sulfonylureas - GLP1 ar - SGLT2 inhibitors	46.7% (*n* = 7) 26.7% (*n* = 4) 13.3% (*n* = 2) 0 0	46.7% (*n* = 7) 20% (*n* = 3) 0 26.7% (*n* = 4) 20% (*n* = 3)	-
Cortical volume (cm3)	438.2 ± 38.5	433.5 ± 34.8	n.s.
Subcortical volume (cm3)	54.4 ± 4.5	52.7 ± 4.6	0.001

Data are presented as *n* (%), mean±SD, or median (IQR), as appropriate. For group comparisons, Student’s *t*-test (continuous variables with normal distribution), Mann–Whitney *U*-tests (continuous variables without normal distribution), and Fisher’s exact test (qualitative variables) were performed.

BMI, body mass index; DPPIV, dipeptidyl peptidase IV; GLP1 ar, glucagon-like peptide 1 agonist receptor; SGLT2i, sodium-glucose transporter 2; MAG, mean absolute glucose change; MAGE, mean amplitude of glycemic excursion; CoV, coefficient of variation.

**Figure 1:**
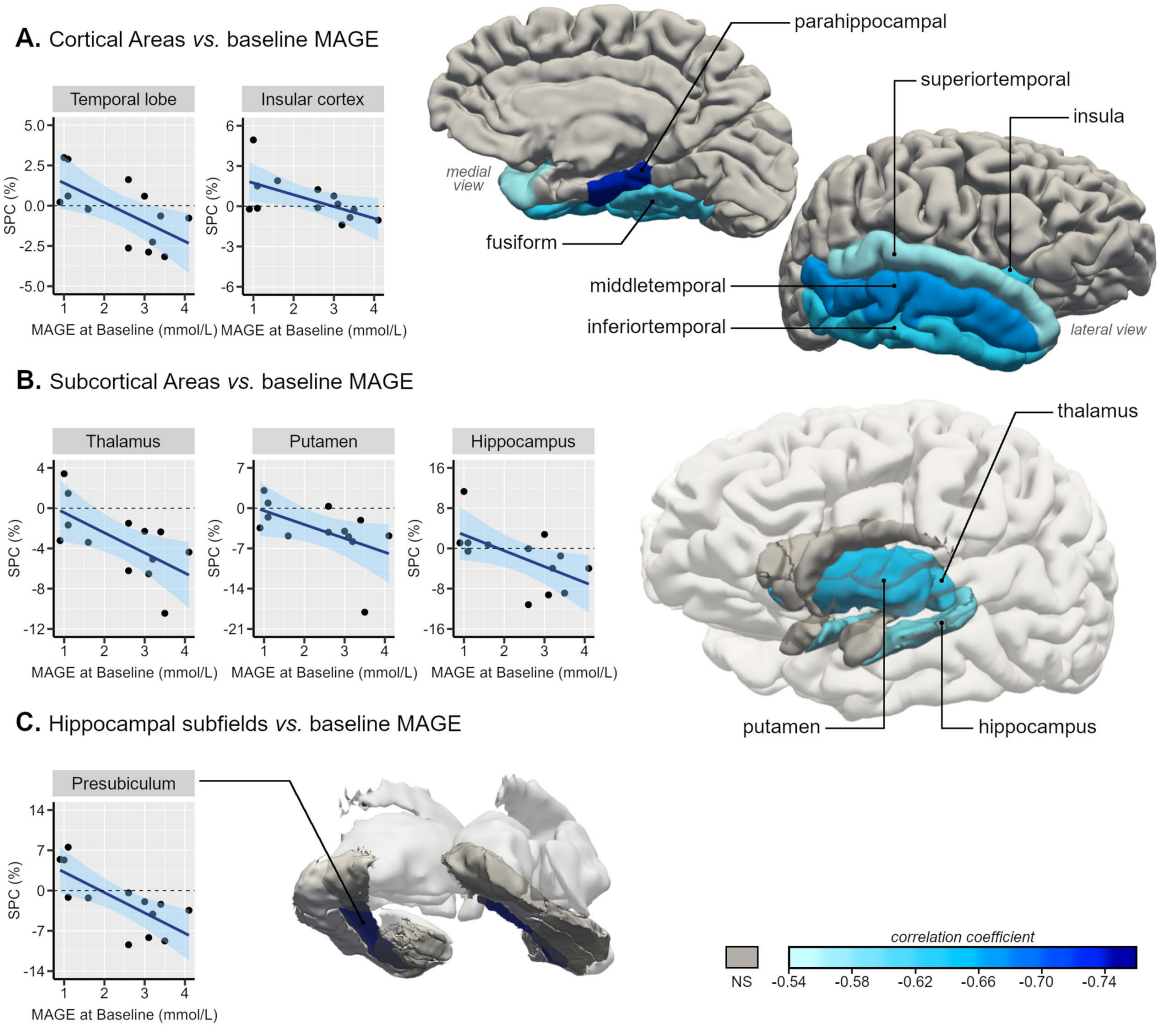
Representative 3D images of the parcellated and segmented brain regions assessed using FreeSurfer. Color intensity corresponds to the correlation coefficient (from −0.76 to −0.54) of the statistically significant associations between baseline MAGE (mmol/L) and symmetrized percent change (SPC, %) of GM volumes between Visit 1 and baseline of type 2 diabetes individuals in **(A)** cortical areas, based on the DKT40 classifier atlas, **(B)** subcortical areas, and **(C)** hippocampal subfields. Scatterplots with line of best-fit with (shaded) 95% confidence band for each region-of-interest (ROI) are also presented.

There were statistically significant strong negative correlations between glycemic variability metrics in baseline (*n* = 13) and temporal lobe SPC: *ρ* = −0.80 for M-value with temporal lobe SPC, *p* < 0.001; *r* = −0.68 for MAG with temporal lobe SPC, *p* = 0.011; *r* = −0.65 for MAGE with temporal lobe SPC, *p* = 0.016 (Figure 1A). Significant negative correlations were observed between variability metrics and insula’s SPC: *ρ* = −0.67 for MAG with insula SPC, *p* = 0.013; *ρ* = −0.63 for MAGE with insula SPC, *p* = 0.022 (Figure 1A).

Concerning the impact of baseline glycemic variability on temporal lobe SPC, a linear regression analysis showed that, for each increase of 1 mmol/L on MAGE value, the SPC of the temporal lobe decreases on average by 1.2% (higher atrophy rate).

**Figure 2:**
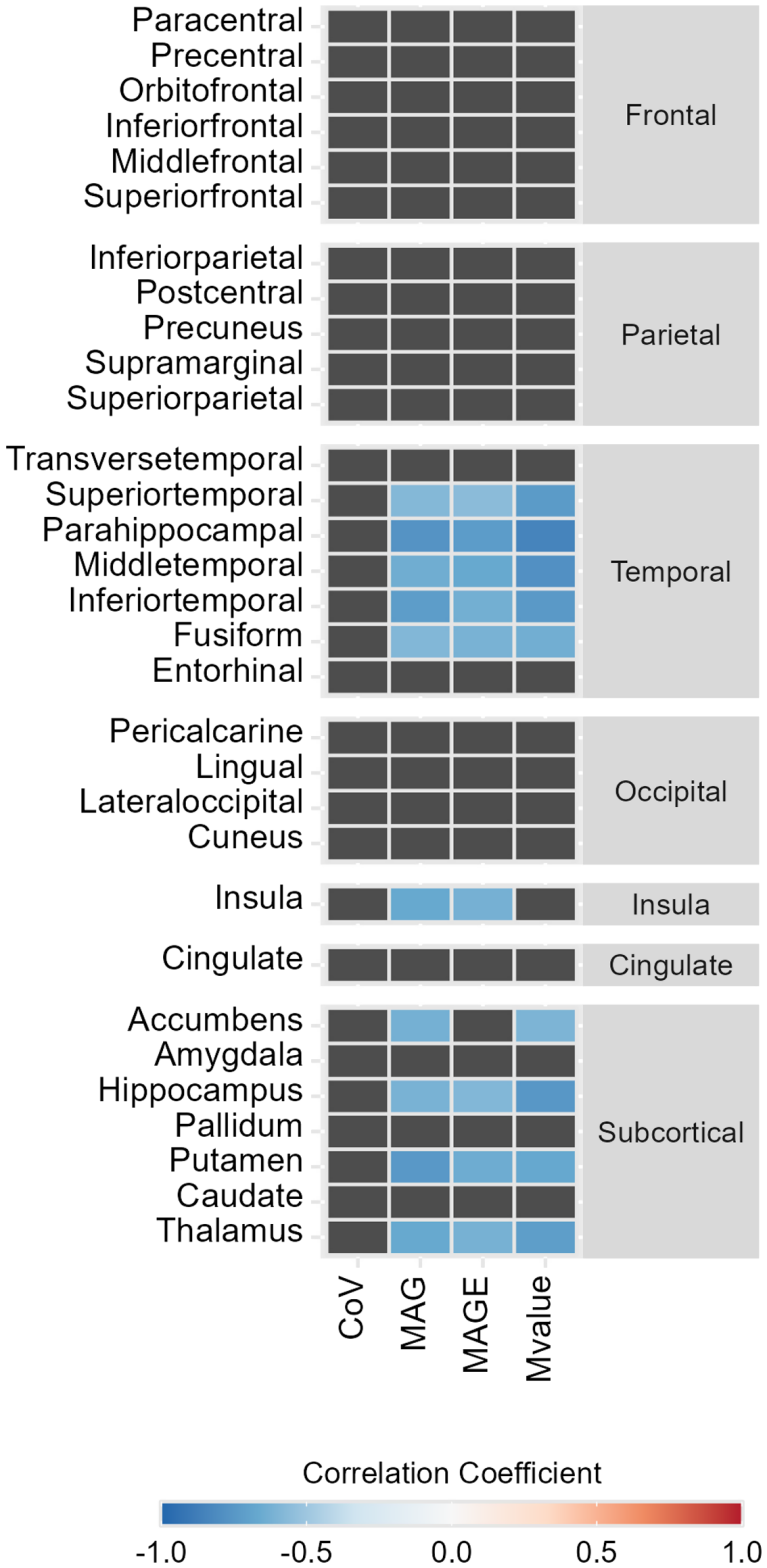
Heat map representing the statistically significant correlations between symmetrized percent change (SPC, %) of GM volumes between Visit 1 and baseline and glycemic variability metrics, in type 2 diabetes individuals. Color represents the strength and the direction of the correlation coefficient (−1, blue; 0, white; 1, red).

Within the temporal lobe, we found a robust pattern of statistically significant negative correlations between GV metrics and temporal lobes’ regions SPC, these correlations varied between *r* = −0.56 [CI_95%_ (−0.85;-0.02)] for the superior temporal gyrus SPC with MAGE and *ρ* = −0.85 [CI_95%_ (−0.96;-0.54)] for parahippocampal gyrus SPC with M-value (Figures 1A, 2). Regarding the correlations between GV metrics in V0 (*n*= 13) and subcortical longitudinal volumes variation (Figures 1B, 2), we found statistically significant negative correlations between: MAG and the thalamus (*r* = −0.62; *p* = 0.023), hippocampus (*r* = −0.62; *p* = 0.025), putamen (*ρ* = −0.75; *p* = 0.003), and accumbens (*ρ* = −0.63; *p* = 0.022); MAGE and thalamus (*r* = −0.64; *p* = 0.024), hippocampus (*r* = −0.59; *p* = 0.034), and putamen (*ρ* = −0.65, *p* = 0.017); M-value and thalamus (*ρ* = −0.72, *p* = 0.006), hippocampus (*ρ* = −0.75, *p* = 0.003), putamen (*ρ* = −0.67, *p* = 0.012), and accumbens (*ρ* = −0.60, *p* = 0.029). These findings remained significant after the *p*-value Benjamini-Hochberg correction.

A detailed analysis of the correlations with the hippocampal subfields’ longitudinal volumes variation was also performed, showing significant negative correlations only in the presubiculum SPC with M-value (*ρ* = −0.86; *p* < 0.001), MAG (*r* = −0.74; *p* = 0.004), and MAGE (*r* = −0.73; *p* = 0.005) (Figures 1C, 2).

The correlations between the (sub)cortical SPC and classical laboratory parameters (including HbA1c and fasting glucose) or clinical characteristics were not significant for either of the visits.

**Figure 3:**
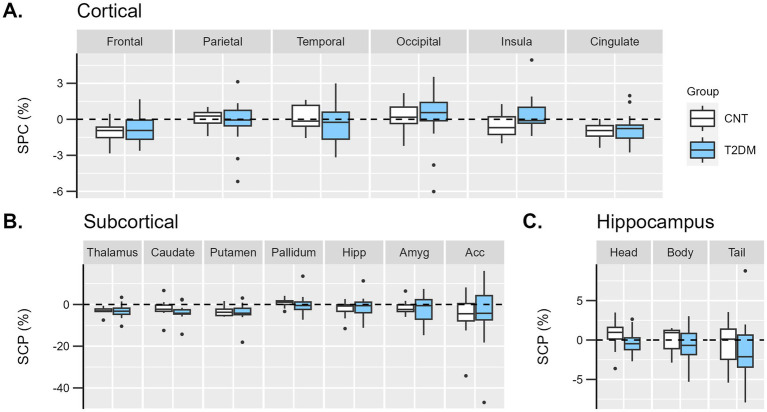
Boxplots representing the distribution of individual symmetrized percent change (SPC, %) of GM volumes between Visit 1 and baseline in diabetes (light blue) and control (white) groups in **(A)** cortical lobes, **(B)** subcortical, and **(C)** hippocampus regions. Hipp, hippocampus; Amyg, amygdala; Acc, accumbens.

A longitudinal assessment of brain atrophy in T2DM vs. controls was performed. Regarding the cortical GM volumes, a repeated measures ANOVA was performed using the lobe’s SPC as a within-subjects effect and group as a between-subjects effect. No statistical interaction effect was found: F_GG_ (2.5, 63.5) = 1.5; *p* = 0.224 (Figure 3A).

Similarly, in the subcortical analysis, a repeated measures ANOVA was performed using the subcortical region’s SPC as a within-subjects effect and group as a between-subjects effect. No statistical interaction effect was found: F_GG_ (1.8, 44.9) = 0.2; *p* = 0.803 (Figure 3B).

Concerning the largest areas of the hippocampus (head, body, and tail), no statistical interaction effect was found: F_GG_ (1.5, 36.4) = 0.03; *p* = 0.934 (Figure 3C).

## 4 Discussion

This study demonstrated strong negative correlations between GV metrics and SPC of regional GM volumes over 7 years in the temporal lobe, insula, and subcortical structures such as the hippocampus, thalamus, accumbens, and putamen of T2DM individuals.

GV metrics are similar in both visits, suggesting that T2DM individuals maintain the same pattern of short-term variability, even though the HbA1c improves from baseline to follow-up visit. Therefore, we used baseline (Visit 0) GV metrics in correlation with brain volumes, exploring the concept of metabolic legacy on the brain (Folz and Laiteerapong, 2021). Our results are consistent with the findings that GV is an independent risk factor for the development of diabetes-related complications (Ceriello et al., 2019) and consequently could have a structural and functional impact on the diabetic brain, mediated by cerebrovascular disease and neurodegeneration. Glucose fluctuations contribute to several damaging pathways mediated by oxidative stress, independently of chronic hyperglycemia (Chang et al., 2012; Torimoto et al., 2013), which could represent a possible conceptual explanation of our results demonstrating the impact of GV on brain volume, independently of HbA1c.

In the only published longitudinal study assessing the indirectly estimated influence of time-specific multi-scale glycemic variability on cognitive impairment and GM atrophy, Cui et al. reported that T2DM individuals with greater glycemic variability had less GM volume in cingulate, hippocampal, middle and inferior temporal gyrus and insula but higher GM volume on supramarginal, left angular, and left middle orbitofrontal gyrus, independently from HbA1c (Cui et al., 2014). Our findings of regionally specific susceptibility to GV identified the temporal lobe, insula, thalamus, hippocampus, accumbens, and putamen as the most affected regions in individuals with T2DM over time, independently from HbA1c. These results might be clinically relevant, since the thalamus stands out as an important subcortical hub in the brain, projecting fibers to nearly the whole cortex, and its volume corresponds to cognitive performances, motor task behaviors, and verbal memory (Hwang et al., 2017). The hippocampal-thalamic-temporal connectivity plays an important role in processing emotions, language, attention, and memory (Behrens et al., 2003). Furthermore, central regions related to habit formation and reward, such as the dorsolateral striatum (putamen) and the nucleus accumbens (Arias-Carrión et al., 2010), and interoceptive processing, such as the insular cortex (Chen et al., 2021), appear to be specifically susceptible to metabolic control. The habit-learning, reward, and prediction-error circuits allow us to update our predictions in response to a mismatch between the actual and expected outcome of an action. This adaptive learning can dynamically direct our choices toward optimal behavior (Diederen and Fletcher, 2021). This strongly suggests that this will have to be considered in patient education programs. In a clinical setting, this could indeed be crucial for a person living with diabetes to adjust an insulin bolus, to prevent a hypoglycemic episode, to select the appropriate nutritional and exercise plan, and to promote better treatment adherence. By hypothesis, glycemic variability may provide a prediction error signal of dopaminergic regulation in T2DM and illustrate how metabolic signals can act as neuromodulators of adaptive behavior. Our results highlight the putative influence of GV in these cognitive processes, even in neurologically preserved T2DM individuals, demonstrating the contribution of GV metrics to the underlying mechanisms of diabetes-related brain atrophy. Among the various factors that might trigger direct neural damage, brain insulin resistance might be a significant contributor, as insulin receptors are selectively distributed in the hippocampus and cerebral cortex (Beddows and Dodd, 2021). Reduced insulin transport across the blood–brain barrier and cerebral insulin resistance in areas of high receptor concentrations may lead to preferential atrophy in these areas.

We applied a (semi) automated longitudinal processing stream to segment MRI images, allowing for more unbiased and sensitive estimations of the SPC of volumes between two time points (Reuter et al., 2012). This pipeline reduces the random variability sources associated with longitudinal analysis by defining a within-subject template and producing more robust and consistent segmentations across time points (Brown et al., 2020). Curiously, in the temporal lobe, the T2DM group had wider dispersion of SPC values, revealing that the GM atrophy in the 7-year follow-up period had a wide variation between subjects, contrary to the control group, which showed homogeneity in temporal SPC over time. In earlier longitudinal studies, T2DM was associated with a 20–50% greater rate of total brain volume over 3–5 years compared with controls (de Bresser et al., 2010; van Elderen et al., 2010). Espeland et al. found a non-significant trend in total brain volume variation of T2DM women over 4.7 years (Espeland et al., 2016). No regional assessment was performed in any of these studies.

Some limitations to our study may arise from the small sample size as, of the initial 170 participants of Visit 0, only 29 agreed to participate in Visit 1. We experienced difficulties in adhering to the follow-up visit, which was related to the COVID-19 pandemic restrictions. Moreover, since data collection started in 2012, the accessibility to real-time or retrospective continuous glucose monitoring devices was low. Therefore, we used 7-point blood glucose sampling, obtained throughout 24 h, for 3 consecutive days. Nevertheless, since the individuals with T2DM selected for Visit 0 of our study were admitted to the Endocrinology ward, blood glucose was measured by well-trained nursing staff, providing data reliability. The Visit 1 blood glucose sampling records were self-reported and showed consistency with the baseline data.

Regarding the comparison of SPC of GM volumes between patients with T2DM vs. controls no significant differences were observed, possibly due to insufficient statistical power.

To the best of our knowledge, this is the first prospective study to examine the influence of intra-daily glucose fluctuations on regional (sub)cortical GM volumes, specifically on areas involved in habit-learning, reward, and prediction-error circuits. Larger longitudinal studies are needed to substantiate our results and emphasize the importance of glycemic variability in clinical practice, as it shows direct correlation with micro- and macrovascular complications and a neural impact in T2DM individuals.

## Data Availability

The raw data supporting the conclusions of this article will be made available by the authors without undue reservation.
